# Melanoma awareness and prevention among latinx and non‐latinx white adults in urban and rural California: A qualitative exploration

**DOI:** 10.1002/cam4.5457

**Published:** 2022-11-25

**Authors:** Rachel J. Mesia, Patricia Rodriguez Espinosa, Hayden Hutchison, Nadia Safaeinili, Laurel J. Finster, Vijaytha Muralidharan, Beth A. Glenn, Robert W. Haile, Lisa Goldman Rosas, Susan M. Swetter

**Affiliations:** ^1^ Stanford Cancer Institute, Stanford University School of Medicine Stanford California USA; ^2^ Stanford University School of Medicine, Office of Community Engagement Stanford California USA; ^3^ Cancer Research Center for Health Equity, Cedars Sinai Medical Center Los Angeles California USA; ^4^ Department of Dermatology/Cutaneous Oncology Stanford University Medical Center Stanford California USA; ^5^ Veterans Affairs Palo Alto Health Care System, Dermatology Service Palo Alto California USA; ^6^ UCLA Fielding School of Public Health, UCLA Kaiser Permanente Center for Health Equity Los Angeles California USA

**Keywords:** health behaviors, health disparities, Latino/a/x, melanoma, rural disparities

## Abstract

**Background:**

Melanoma mortality rates in the US are highest among older men, individuals of lower socioeconomic status (SES), and people of color. To better understand these inequities, a qualitative exploratory study was conducted in Northern and Southern California to generate knowledge about barriers and facilitators of awareness, prevention, and early detection of melanoma in lower SES Latinx and non‐Latinx White (NLW) individuals living in urban and semi‐rural areas.

**Methods:**

Nineteen focus groups were conducted (*N* = 176 adult participants), stratified by race/ethnicity (Latinx, low‐income NLW), geography (semi‐rural, urban), and language (English and Spanish). Inductive and deductive thematic analysis was conducted, and the findings were organized using the socioecological model framework: individual, interpersonal, community, and health system/policy levels.

**Results:**

Four socioecological themes describe how key factors affect knowledge, perceived risk, preventive behaviors, and melanoma screening. Individual level findings revealed that many participants were not familiar with melanoma, yet were willing to learn through trusted sources. Having brown or darker skin tone was perceived as being associated with lower risk for skin cancer. Interpersonally, social relationships were important influences for skin cancer prevention practice. However, for several Latinx and semi‐rural participants, conversations about melanoma prevention did not occur with family and peers. At the community level, semi‐rural participants reported distance or lack of transportation to a clinic as challenges for accessing dermatology care. Healthcare systems barriers included burdens of additional healthcare costs for dermatology visits and obtaining referral.

**Conclusions:**

Varying factors influence the awareness levels, beliefs, and behaviors associated with knowledge, prevention, and early detection of melanoma among low‐income Latinx and NLW individuals and in semi‐rural areas. Results have implications for health education interventions. Navigation strategies that target individuals, families, and health care settings can promote improved prevention and early detection of melanoma in these communities.

## Graphical abstract

Disparities in melanoma outcomes appear to be associated with varying factors that influence community knowledge, prevention, and early detection of melanoma. This qualitative study can help inform the design of effective and culturally relevant melanoma prevention and early detection strategies among low‐income Latinx and non‐Latinx White adults across rural and urban regions.

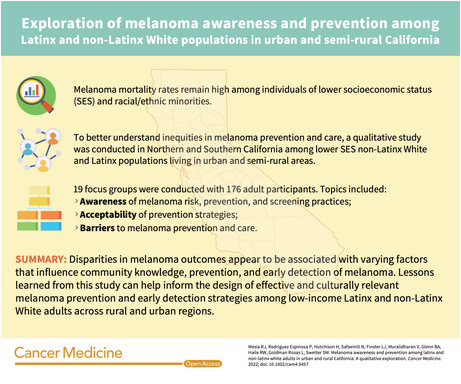

## 1 INTRODUCTION

Melanoma is preventable by reducing harmful ultraviolet radiation exposure^1^ and highly curable if detected in its earlier stages,^2^ resulting in lower morbidity, mortality, and healthcare costs. In the United States (US), melanoma mortality rates have declined by nearly 18% since 2014 in non‐Latinx White (NLW) individuals^3^; however, similar trends are not apparent in those of lower socioeconomic status (SES), including the Latinx community, and those living in rural areas.^4^, ^5^, ^6^, ^7^, ^8^, ^9^, ^10^, ^11^ This may be attributed to less access to the information and services that are critical for preventing, detecting, and treating melanoma.

The melanoma burden is increasing in Latinx adults in California, who represent the largest ethnic group in the state, at 39%,^12^ and typically present with more advanced disease.^9^, ^13^, ^14^ While US melanoma incidence rates remain low among Latinx adults compared to NLWs (4.6 vs. 24.9 per 100,000 from 2012 to 2016), melanoma mortality is higher compared with other non‐white racial and ethnic groups.^13^, ^15^ Differences in primary melanoma location (leg/hip/foot) and clinicopathologic subtypes (acral and nodular) in Latinx adults compared with NLWs tend to hamper early recognition.^9^, ^15^, ^16^, ^17^ Likewise, physician‐ and self‐skin examination is reported at lower rates in Latinx adults compared to NLW adults.^18^

Similar inequities are apparent in rural and semi‐rural communities. California's rural and semi‐rural populations reflects US rural populations, which are primarily composed of NLW and Latinx individuals, who tend to have lower SES.^19^ Limited income and education and less access to health services correlates with thicker melanoma diagnosis and increased mortality risk.^4^, ^5^, ^6^, ^7^, ^8^, ^9^, ^10^, ^11^ Likewise, a lower density of dermatologists and oncologists in rural regions correlates with increased melanoma mortality and is^20^, ^21^ compounded by poverty, large elderly populations, overall poorer health and distance to specialized clinics in metropolitan areas.^14^ Moreover, the growth of racial and ethnic minority and immigrant populations in rural and semi‐rural communities highlights the need to understand specific cultural and language needs as well as socioeconomic and geographic factors that may hinder melanoma early diagnosis and treatment.^22^, ^23^

As very little is known about how best to prevent and reduce the melanoma burden in these communities, we conducted a series of focus groups with low‐income NLW and Latinx participants, the two largest racial/ethnic groups in the region, residing in Northern and Southern California to better understand barriers and facilitators of prevention and early detection strategies. This exploratory qualitative study is a first step in designing effective and culturally relevant melanoma prevention strategies in these high‐risk populations.

## 2 MATERIALS AND METHODS

### 2.1 Population and settings

Using a purposive sampling strategy and leveraging community‐based stakeholder relationships, participants were recruited through community leaders, Facebook, Craigslist, churches, community centers, libraries, local stores and cafes, cultural groups, hospital‐based health education programs, and federally qualified health centers to participate in a series of focus groups. Focus groups were designed to be homogeneous according to region (Northern or Southern California), community type (semi‐rural vs. urban), self‐reported ethnicity (Latinx or non‐Latinx white), and language (English or Spanish). Participants self‐reported their demographics, including: annual household income and the number of individuals supported by this income, English language fluency level (i.e., native English speaker, fluent English, speak some English, do not speak any English), and race and ethnicity or cultural group(s). While recruitment efforts focused on the White or Hispanic/Latinx population, interested individuals from other racial and ethnic groups who met eligibility requirements (i.e., able to consent, over 18 years of age, able to speak English and/or Spanish) could participate in the study. The study was approved by the Institutional Review Boards of both Stanford University and Cedars‐Sinai Medical Center. All participants gave their informed consent prior to their study involvement. Participants were served dinner and received a $30 gift card after the focus group to thank them for their participation.

### 2.2 Focus group protocols

In 2019, trained facilitators conducted in‐person focus groups (except one virtual interview), which included 7–9 participants on average and lasted approximately 2 hours. Facilitators who were bilingual in English and Spanish led focus groups with Spanish speakers. A semi‐structured interview guide, partially informed by the Health Belief Model (HBM)^25^ and the Socioecological Model, was used by facilitators to maintain a consistent framework for asking questions related to health beliefs and socioecological factors impacting health behaviors. Topics assessed during focus groups included the following: (1) participants’ awareness and perceptions of melanoma risk, prevention, early detection and screening practices; (2) acceptability of primary and secondary prevention strategies in their respective community, and (3) barriers and facilitators of engagement in melanoma prevention and care. All focus group discussions were audio‐recorded and transcribed verbatim.

### 2.3 Data analysis

Transcripts were independently coded by two members of the research team in NVivo 11 using a hybrid deductive and inductive approach.^24^ A‐priori codes included theory‐based codes informed by the HBM^25^ and codes based on the interview guide. Debriefing notes were used to cross‐validate the initial coding scheme. Emerging themes were later organized using the socioecological framework.^26^

Coded transcripts were reviewed by a member of the research team (NS) monthly to ensure continued coding consistency between the two coders. Where consensus could not be met, the researchers consulted a third qualitative expert (PRE) to reach consensus. Post‐coding analysis included additional review of the data to extract group‐level similarities and differences by geographic location, self‐identified racial/ethnic group, and various combinations of both.

## 3 RESULTS

We conducted 19 focus groups with a total of 176 participants. Most participants were female (77%), 51% were non‐native English speakers, 59% self‐identified as Latinx/Hispanic/other Hispanic ethnicity, and 40% had Medi‐Cal or state insurance coverage (Table 1). Below we summarize focus group findings within four levels of the socioecological framework: individual, interpersonal, community and system/policy.^25^ Within each level, we describe findings in relation to the Health Belief Model constructs that emerged as most relevant.

**TABLE 1:** Demographic background by geographic region, *N* = 176

	Urban^a^, *N* = 92	Semi‐Rural^b^, *N* = 84
Number of focus groups		
Northern CA	7	4
Latinx/Hispanic^c^	3	2
Non‐Latinx White (NLW)	4	2
Southern CA	4	4
Latinx/Hispanic^c^	2	2
Non‐Latinx White (NLW)	2	2
Race/ethnicity		
Latinx/Hispanic^c^	51 (55%)	52 (62%)
Non‐Latinx White (NLW)	25 (27%)	22 (26%)
Other race/ethnicity^d^	3 (3%)	4 (5%)
No response	13 (14%)	6 (7%)
Gender		
Female	64 (70%)	71 (85%)
Male	28 (30%)	13 (15%)
Nativity		
Foreign‐born	53 (58%)	39 (46%)
English fluency		
Native English speaking	35 (38%)	40 (48%)
Fluent English speaking	25 (27%)	20 (24%)
Some English speaking	24 (6%)	20 (24%)
Do not speak any English	7 (8%)	4 (5%)
Healthcare insurance status		
No insurance coverage	10 (11%)	8 (10%)
Employer‐sponsored	24 (26%)	12 (14%)
Self‐purchased	3 (3%)	6 (7%)
Spousal coverage	5 (5%)	6 (7%)
Medi‐Cal/State	33 (36%)	38 (45%)
Medicare	6 (7%)	10 (12%)
Other type of insurance	9 (10%)	3 (4%)
Highest level of education completed		
Grade school or less	15 (16%)	9 (11%)
Some high school	12 (13%)	7 (8%)
High school/GED	8 (9%)	23 (27%)
Some College/Technical school	26 (28%)	32 (38%)
College graduate	21 (23%)	9 (11%)
Graduate degree	10 (11%)	4 (5%)
Marital status		
Do not have a partner	33 (36%)	17 (20%)
Divorced	6 (7%)	7 (8%)
Married or Living with my partner	38 (41%)	42 (50%)
Have a partner, not living together	7 (8%)	3 (4%)
Separated	5 (5%)	3 (4%)
Widowed	2 (2%)	11 (13%)
Employment status		
Full‐time	40 (43%)	13 (15%)
Part‐time	23 (25%)	16 (19%)
Homemaker	10 (11%)	14 (17%)
Unemployed, not looking	1 (1%)	1 (1%)
Unemployed, currently looking	8 (9%)	5 (6%)
Retired or disabled	7 (8%)	24 (29%)
Other	2 (2%)	9 (11%)
Children		
No	35 (38%)	15 (18%)
Yes	57 (62%)	69 (82%)
Annual household income (before tax deductions)		
$25,000 or less	31 (34%)	37 (44%)
$25,001 ‐ $75,000	33 (36%)	35 (42%)
$75,001 ‐ $150,000	13 (14%)	7 (8%)
$150,001 or more	6 (7%)	1 (1%)
No response	7 (8%)	4 (5%)

Abbreviation: GED, tests of general educational development.

^a^ Urban: The term “urban” is used to describe a metropolitan community with: (1) dense urban/suburban populations; (2) small land ratio of ranchland, farmland, or wilderness; (3) multiple resources and industries available to its populations.

^b^ Semi‐rural: Rural geography and populations are defined differently by federal agencies and organizations. The term "semi‐rural" is used to describe geographic areas that: (1) are not considered rural according to any of the federal definitions; (2) are further distant from metropolitan regions; (3) have small population clusters dependent on a limited number of industries.

^c^ Latinx/Hispanic: The term “Latinx” alongside “Hispanic” is used to represent a broader range of self‐identities within the Latino/a/x populations, with the use of “x” to embody gender diversity.

^d^ Other race/ethnicity: Indicates participants whose ethnic or cultural group(s) response was not in the White or Hispanic/Latinx category. Other racial/ethnic categories: Asian, African American/Black, and American Indian/Native American.

### 3.1 Individual: Knowledge, attitudes, perceived barriers

#### 3.1.1 Knowledge

Many focus group participants expressed little to no knowledge about melanoma and other skin cancers. Across the various groups, there was confusion or limited understanding about sunscreen use, the safety of sunscreen ingredients, and the meaning of sun protection factor (SPF) values. Participants expressed that they and their communities knew very little about melanoma due to lack of publicity and conversation (Table 2 (1c)). Some participants perceived skin cancer as being a less severe cancer condition because a physician “*can just cut it out and [you’ll] be ok*”. Female participants who considered themselves or family members as having lighter skin or sun sensitivity tended to be knowledgeable about the harmful effects of the sun but were unfamiliar with melanoma.

**TABLE 2:** Major themes organized by levels of the socioecological model

Socioecological level theme	Exemplary quotes	Urban		Semi‐rural	
		Latinx	NLW	Latinx	NLW
Individual					
1a. Knowledge facilitator: Trust in institutions and physicians	*“Organize, create information groups with a doctor to give us information.”* (*Latinx focus group, urban*)	•	•	•	•
1b. Knowledge facilitator: Access to information	*“They believe the minister will help them… sometimes I don't have the answer. But the thing is they come [to] me… And start giving me all the symptoms.”* (*Latinx focus group, urban*)	•			
1c. Knowledge barriers: Access to information	*“It just is not in the spotlight as much, so I just kind of put it in the back of my mind and say, ‘Well, whatever’. I just don't worry about it”* (*NLW focus group, urban*).	•	•	•	•
	*“…Probably nobody pays too much attention to it, or just they think it's not as risky the other types of cancers…”* (*Latinx focus group, semi‐rural*)				
1d. Attitudes facilitator: Parents are motivated to practice primary prevention to protect their children	*“…My daughter's so vigilant about sunscreen, hats, being covered up…I've always followed her direction because ultimately she's the mom…My daughter is way over here on how she grew up.”* (*NLW focus group, semi‐rural*)	•	•	•	•
1e. Attitudes barriers: Mainstream beliefs, Cultural identity	*“Hispanic Latinos…… why and my color of skin will protect me… maybe that's one of the excuses… they say, “No because I'm brown.”* (*Latinx focus group, urban*)	•	•	•	
	*“I didn't use sunscreen and I tanned really dark. And my children are multi‐cultured…They're real dark. But my son, whom at the time was seven, got too much sun that I didn't know because of his skin being so dark, but then his skin started rolling off.”* (*NLW focus group, urban*).				
1f. Attitudes barriers: Gender norms	*“Men on the other hand are more like knuckle dragging Neanderthals. So, we don't, at least I don't, take it as a top concern… We're probably not as educated, or at least we don't care as much.”* (*NLW focus group, urban*)	•	•	•	
	*“…Men are stubborn and they just, ‘Oh, okay. It's just a little hurt here or pain…I'm good.”* (*Latinx focus group, semi‐rural*)				
1g. Perceived barriers: Competing priorities	*“Poverty also plays a part. As my mother says: ‘Here you work one day to buy a [block of cheese] and two gallons of milk,’ that's what you earn for a workday. People will not stop buying food to buy sunscreen.”* (*Latinx focus group, semi‐rural*).	•		•	•
	*“…Some people they don't have any money for food, so how they can have money for buying that stuff [sunscreen].”* (*Latinx focus group, semi‐rural*)				
	*“No, I'm not paying for that. I mean I'm worried about the diabetes…about women's health…[Dermatology] falls into luxury as far as health…”* (NLW focus group, urban)				
1h. Perceived barriers: Primary physicians perceived as less knowledgeable	*“…I can ask my doctor, but sometimes my doctor doesn't even know what to refer me to or where if he's not too savvy with that situation then, we are lost.”* (*Latinx focus group, semi‐rural*)	•		•	•
	*“…More PCPs…overlook a lot of things because they are seeing…a lot of patients in the day and they don't necessarily specialize in any one type of thing…I cannot say I've ever had a primary care doctor actually check my skin for any type of growths or anything like that. It's always just like your vitals.”* (*NLW focus group, semi‐rural*)				
1i. Perceived barriers: Primary physicians perceived as being inconvenienced about patient*'*s skin concerns	*“And I almost feel like that's why my doctor doesn't say anything to me, because they are like, ‘Oh, I don't want to get started on that and be the doctor that has to look at every single one of his freckles.’ That's how I feel.”* (*NLW focus group, semi‐rural*)	•		•	•
	*“Nurse practitioners I think care a little more. PCPs are just like whatever. Next.”* (*NLW focus group, semi‐rural*)				
1j. Perceived barriers: Limited or lack of trust in the healthcare system*'*s billing of patients	*“Go to the dermatologist, ‘Oh, it's nothing. Don't worry about it.’ Meanwhile, they are charging their insurance every time we went.”* (*NLW focus group, semi‐rural*).		•		•
	*“That you all always concerned or tried to get more money from me or is it really that I need it right now. So, here especially in this country, that's probably a really, really a big problem.”* (*NLW focus group, urban*)				
Interpersonal					
2a. Facilitators: Family member or peer influence and reinforcement to practice primary and/or secondary prevention behaviors	*“My wife… my daughters, my son, everyone, they all protect themselves… By force… All the time [my wife] simply smears [sunscreen] on me.”* (*Latinx focus group, semi‐rural*).	•	•	•	•
	*“My mom would be like, ‘If we are going to go to the beach, going to go to the lake, put on sunscreen so you don't get a sunburn’…She didn't [make] me wear it because of skin cancer.”* (*NLW focus group, semi‐rural*)				
	*“…Maybe because of the cultural difference – Americans put sunscreen on their children even if they are just going to school. Now I do that too with my little girl, because she does her school break in the sun. And I do it for myself too, but because I already know maybe a little more about the risks of not doing it.”* (*Latinx focus group, semi‐rural*)				
	*“My father has had it on his nose and on his cheek…I've worked outdoors most of my life…and I was told to watch for moles that change color, and spots, a sore or something…I've always tried to wear long sleeves and a hat…Pretty much the family…we all kind of knew…Not so much the technicalities or the details of melanoma, but just watch out…”* (*NLW focus group, semi‐rural*)				
2b. Facilitators: Peers in agricultural and outdoor occupations practice primary prevention behaviors	*“…And towards the 90 s, 2000 s, a lot of the women would put bandanas and hats and long sleeves and gloves. I never asked, but I presumed that was a reason because of skin cancer, sun, all day long…In the South Valley, Kern County and the harvesting trees…a lot of the men wore long sleeves… keeps the sun off you and the straw hat. I know a lot of people in that field, I guess you could say, are aware of it* (*NLW focus group, semi‐rural*)			•	•
2c. Facilitators: Community‐based settings are trusted places for obtaining information	*“In Merced, if you are in a hospital, there they do give information about different things like taking care of your heart, different things…You can put a workshop there or other things like that.”* (*Latinx focus group, semi‐rural*)	•	•	•	•
	*“…The Latino communities [are] really religious so that'll be another good way to get the information to people. By talking to the pastors, the priests…because everyone, everyone pays attention to someone that has like an authority*. (*Latinx focus group, urban*)				
2d. Barriers: Melanoma, skin cancer, or the importance skin cancer prevention are not acknowledged among family or friends.	*“…Especially in the Latino community, we don't see it as a big occurrence. The most common diseases for Latinos are like, diabetes or Crohn's… or high blood pressure, but skin cancer, to be honest, none of our close friends or family that we know of have skin cancer, so… it doesn't really concern us.”* (*Latinx focus group, urban*)	•	•		
	*“We don't talk about skin cancer. We don't talk about … until it's the very end…They kind of dismiss the symptoms until the very last minute. And I don't think I can remember a time where my mom would say, “Put on sunscreen.”* (*Latinx, urban*)				
Community					
3a. Barriers: Limited or lack of transportation options for travel to healthcare visits	*“Problems, I did not know that there were busses, I just learned that there were busses.”* (*Latinx focus group, semi‐rural*)			•	•
3b. Barriers: Far distance to available medical specialist	*“There are no specialists here. You have to go to San Jose…You have to leave the area.”* (*Latinx focus group, semi‐rural*)			•	•
	*“Great service, I went with my mom, but even during the week it takes 2–3 hours to get there…Well, I don't drive…”* (*Latinx focus group, semi‐rural*)				
3c. Barriers: Lack of community‐wide information dissemination and communication about skin cancer prevention	*“I feel like the community doesn't even ask, because I have so many questions, but I don't want to ask you. So, seeing that there's a lack of communication between patient and doctor, the doctor should be aware that if maybe the patient is not maybe comfortable enough to say it, or maybe shy to say it, or doesn't want to bring it up…”* (*Latinx focus group, urban*)	•	•	•	•
	*“I don't think I know anything else being told to people besides put on your sunscreen…because most of the people I see around here usually have the burn.”* (*NLW focus group, semi‐rural*)				
Healthcare systems and health policy					
4a. Facilitators: Occupational or workplace requirements include sun protective apparel and/or protocols	*“…She was allowed to wear a cap because she was not to get around the sun. So once the sun hit down, [the employer] put her back to where her post was.”* (*Latinx focus group, semi‐rural*)			•	
4b. Facilitators: Support toward school provisions related to skin cancer prevention	*“[Sunscreen] must be kept and provided in certain places such as schools, where children are exposed [when] running outdoors.”* (*Latinx focus group, urban*)	•	•	•	•
	*“[The school] also give us training to know the risks.”* (*Latinx focus group, semi‐rural*)				
4c. Barriers: Uncertainty about the healthcare system	*“The clinics here, I will say, this is personally because I have seen it in 28 years in this town, the medicine is very behind, the doctors are very behind, there is very bad medical service.”* (*Latinx focus group, semi‐rural*)			•	•
	*“They always change, the doctors are different. I do not have a main doctor, familiar, no because here they are constantly being changed. Now you go, tomorrow they will change it and this doctor doesn't know your information well.”*(*Latinx focus group, semi‐rural*)				
4d. Barriers: Lack of healthcare coverage or out‐of‐pocket healthcare cost deters individuals from seeking healthcare.	*“My brother‐in‐law, he died from melanoma… He always had this big freckle on his neck. It got bigger and he still ignored it because he didn't have health insurance. Then, he finally got a job where he had health insurance. He finally went to a doctor for the first time and they tell him he's got cancer.”* (*NLW focus group, semi‐rural*)	•	•	•	•
	*“[Skin exam] should be included, but sometimes insurance doesn't cover it and the person cannot pay. Some insurances don't cover everything.”* (*Latinx focus group, semi‐rural*)				

*Notes*: The bullet symbol indicates that the socioecological level theme is correlated to the ethnic group and its corresponding community type.

Health information from trusted, preferred sources was cited as important for learning more about melanoma. Medical institutions, local clinics, and physicians were highly preferred and perceived as credible and trusted health information sources (Table 2 (1a)). Yet, several participants reported that education about melanoma prevention and screening from the physician, especially from primary care, did not occur regularly or at all (Table 2 (1h)). Several participants reported being exposed to online skin cancer information, but some participants considered online information hard to parse through or less trustworthy. A family member, friend, or trusted community member were cited as sources for obtaining health advice. For instance, a church pastor explained a viewpoint that faith‐based leaders such as himself were considered trusted sources for health advice within the Latinx community, emphasizing that men preferred faith‐based leaders as a first source of support (Table 2 (1b)).

#### 3.1.2 Attitudes

Skin cancer risk perceptions were influenced by parental role, mainstream beliefs, cultural identity, and/or gender norms. Risk perception influenced the practice of skin cancer prevention behaviors. Participants cited that motherhood influenced skin cancer risk perception and prevention behaviors because of concern for the children's well‐being (Table 2 (1d)). Males and those with darker skin (who do not get sunburned) believed that they were at little or no risk for skin cancer. Many Latinx women and men participants shared, often with cultural identity pride, that their darker or brown skin color was protective against sun damage or skin cancer risk (Table 2 (1e)). Some NLW and Latinx male participants expressed how their concept of masculine identity dissuaded the practice of preventive behaviors that were perceived as a “beauty routine” (Table 2 (1f)). These beliefs shaped the attitudes towards not seeking information about skin cancer or prioritizing skin cancer prevention practices. Moreover, participants in the Latinx group mentioned that Latinx men or themselves were reluctant to seek medical attention for their health concerns until a skin lesion became immediately painful (Table 2 (1f)).

#### 3.1.3 Perceived barriers

Regarding individual obstacles for skin cancer prevention and early detection, the participants identified competing priorities, financial hardships, and concern about primary care physician knowledge related to melanoma screening and referral. Several participants viewed skin cancer prevention products and dermatology services as cost prohibitive, especially for those living in rural areas and among Latinx adults. While many participants felt they would use sunscreen more regularly if it was free or more affordable, some individuals weighed the decision to prioritize expending for “basic needs” over sunscreen (Table 2 (1g)). Dermatology care was generally viewed as a lower health care priority due to competing health concerns and/or additional healthcare costs (Table 2 (1g)), and a few participants from NLW groups alluded to a lack of trust in healthcare billing practices (Table 2 (1j)). Concern regarding a primary care physician's expertise in melanoma screening or guidance was also a common theme, which arose from experiences of skin cancer concerns not receiving attention, being dismissed, or minimized (Table 2 (1j–i)).

### 3.2 Interpersonal: Relationships

The promotion of healthy behaviors related to melanoma prevention was mostly influenced by family members, friends, or peers. Participants reported not talking to peers or family members about melanoma because they either lacked awareness about melanoma or had no personal experience with this disease (Table 2 (2d)). However, social and work relationships influenced the practice of melanoma prevention behaviors. Individuals who did not prioritize skin cancer prevention used sunscreen on themselves or on their children due to familial or peer influence (Table 2 (2a)). Both NLW and Latinx female participants emphasized that family‐shared gender and beauty norms encouraged sun protection behaviors (Table 2 (2a)). For agricultural and outdoor occupations, the workers were exposed to the ongoing practice of many workers wearing sun protective clothing and apparel in their work environment (Table 2 (2b)). Although skin examination was seldomly discussed as a prevention behavior practice, some participants reported that shared perceptions from family, spouse, or friends about the importance of skin awareness led them to practice skin examination (Table 2 (2a)). Among Latinx participants, self‐identified lighter‐skinned young women reported receiving messages about skin protection from female family elders due to their complexion or family health history.

### 3.3 Community: Physical and social environment

Across focus groups, built or physical environment, proximity to resources (e.g., clinics), as well as community cultural and social norms were important facilitators and barriers shared by participants. Lack of transportation, long distance to an available medical specialist, and out‐of‐pocket healthcare cost were discussed, especially in rural areas, as significant barriers for seeking a dermatologist (Table 2 (3a–b)). More rural than urban participants reported facing barriers related to transportation to primary care and medical specialist appointments. Some participants shared that traveling to the nearest dermatologist or other medical specialist could take between one and 4.5 hours each way, due to few specialists located in rural communities.

Many focus group participants viewed melanoma as a topic on which community members did not receive sufficient information in their locality and did not have great concern for (Table 2 (3c)). One participant noted that an urban city outside California where she lived during childhood was a place that heavily promoted information about skin cancer and prevention. However, in her current rural community, she observed that skin cancer awareness was considerably lower, and behaviors toward skin cancer prevention (e.g., sunscreen use, skin checks) differed as well. Other participants of rural communities validated this observation.

### 3.4 Healthcare systems and health policy

#### 3.4.1 Healthcare systems

Many healthcare challenges emerged, including lack of access to dermatologists, high costs, and low prioritization of melanoma screening by primary care providers. Some participants also expressed barriers to receiving dermatology referrals, long wait periods, and the accompanying financial costs. Some participants expressed that being uninsured or the additional healthcare costs deterred them or members of their community from seeking dermatology care (Table 2 (4a)), and that sufficient health insurance coverage was critical to paying for their dermatology care needs. Latinx participants shared additional factors influencing their healthcare access and physician‐patient interactions around melanoma. General distrust of the medical system due to a history of negative experiences included perceptions that their concerns and pain were not always believed or taken seriously by healthcare providers (Table 2 (4b)).

#### 3.4.2 Health policy

Policy or regulations were also perceived as important for health prevention efforts. For example, some individuals who made observations of workers in construction, agricultural, or other outdoor occupations, recommended a regulation around protective clothing and skin protection through their employers (Table 2 (4c)). However, there were some outdoor workers, particularly in the Latinx groups, who did not reference employer provisions for sun protection or sun protective clothing. For school settings, several participants of varying backgrounds expressed opinions of having school provisions on skin cancer prevention education to young students and their parents, as well as requiring schools to supply the students with sun protective items such as sunscreen and hats (Table 2 (4d)). The high value of protecting adolescent well‐being appeared to encourage the discussion of school‐based health policy for sun protection.

## 4 DISCUSSION

**FIGURE 1:**
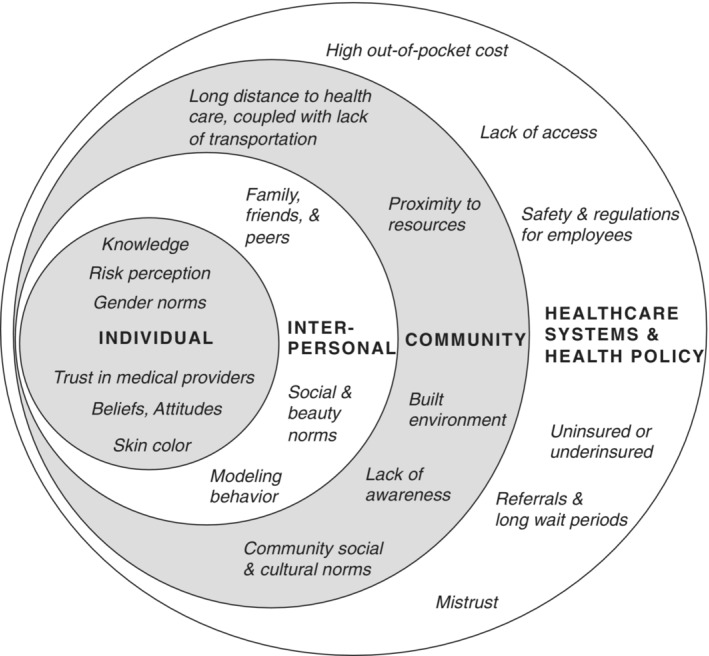
Key factors across the socioecological spectrum: Organized according to socioecological level themes of individual, interpersonal, community, and healthcare systems/health policy, the diagram provides an overview of the main factors affecting awareness, beliefs, and behaviors that were related to the knowledge, prevention, and early detection of melanoma.

Disparities in disease outcomes and gaps in perception of melanoma risk are seen across different races, ethnicities, and geographies. Little is known about how to effectively address melanoma in low‐income, rural, and/or Latinx populations. Our study provides new knowledge that can inform the development and implementation of melanoma control interventions. We identify multiple factors across the socioecological spectrum that facilitate or hinder the participants’ and community's knowledge about melanoma, prevention practices, and access to care for melanoma early detection (Figure 1).

Across race and ethnicity and geographic locations, the participants’ beliefs, risk perception, cultural and gender norms, competing priorities, and self‐efficacy were key facilitators and barriers correlating to health information seeking behaviors, melanoma prevention, and early detection practices. Many participants reported themselves and their community as having little to no knowledge about melanoma, prevention information, and/or perceived risk, particularly among Latinx adults.

Other studies have shown that Black and Latinx adults perceived their skin cancer risk as low,^7^, ^27^, ^28^ reducing performance of skin self‐examination.^6^, ^29^ Rodríguez et al. reported that their Latinx focus group participants felt that “Latin skin” is “resilient,” and darker skin allows for longer sun exposure without negative health repercussions.^28^ A study measuring Latinx lay health workers’ (LHW) confidence levels to conduct self‐examination of the skin found that prior to attending melanoma educational sessions, all LHW participants (*N* = 12) reported being “not at all confident” in conducting skin examinations.^29^ Our qualitative study confirms low‐income, semi‐rural, and Latinx adults’ unfamiliarity with melanoma as a potential serious health problem, and underscores why they lack awareness about monitoring skin changes and the importance of early detection. Our findings support the need for developing additional health education and promotion efforts to effectively reach these groups.

Fostering increased knowledge and preventive behaviors calls for multilevel efforts that can address information dissemination by trusted sources and the incorporation of gender, social, and cultural norms. While physicians were prominently viewed as a trusted source for health information, many participants did not receive skin cancer information from them. Our findings support the need to involve family, peers, and trusted community members, especially in Latinx communities. For instance, sun protection practices were positively influenced by family members or peers.

Melanoma mortality rates and thicker melanomas at diagnosis are higher for males, especially those who are of lower SES, uninsured, and/or persons of color.^30^, ^31^, ^32^ As such, male gender constructs and norms (e.g., masculinity, low risk perception, and help seeking) need to be addressed in messaging and interventions, including the importance of promptly addressing health concerns. Strategies involving reinforcement from family and trusted relationships along with culturally responsive and literacy/language‐appropriate melanoma education are necessary for changing risk perception, initiating preventive behaviors, and promoting self‐efficacy in low‐income, rural, and Latinx groups. Tailored health prevention messages should focus on specific groups based on skin cancer risk, skin color, gender norms, and ethnicity or cultural identities.

Within the healthcare system, lack of access and mistrust were found to be important barriers to melanoma prevention. Low SES, rural residents, and Latinx participants experienced significant challenges with health care access (especially to dermatologists), out‐of‐pocket costs, physician communication, and having limited confidence in their healthcare provider. This may explain why delays in seeking health care occur in populations with limited or no means of affording health services. Prior studies have shown that low SES, underinsured, and uninsured patients are diagnosed with more advanced stages of melanoma, potentially due to delays in seeking medical attention.^7^, ^31^, ^33^, ^34^ Furthermore, for rural participants, the financial burden of healthcare is compounded by unavailable specialty care, lack of transportation, and/or belief that healthcare in their local region is not “high quality.” These obstacles make melanoma prevention and early detection difficult.^35^, ^36^ Finally, poor communication and experiences with providers, reported by Latinx participants, may further deter Latinx individuals from seeking healthcare or openly communicating about health concerns. As a critical part of melanoma control, attention is needed on improving healthcare system access, communication and interactions.

### 4.1 Future directions

The findings from this study have informed the development of a pilot intervention that will test the efficacy of a culturally and linguistically appropriate health education intervention, delivered by trusted messengers such as community health workers, to promote melanoma prevention and early detection alongside health care navigation. Similar efforts that create infrastructure and models for collaboration, such as the Wipe Out Melanoma—California statewide initiative to promote early detection, education, and prevention research among high‐risk populations, should be prioritized.

### 4.2 Study limitations

There are several limitations of this study. Participants represent a convenience sample within each of our target strata, so may not represent the larger community from which they were recruited. Although the focus of our study was specifically to better understand barriers and facilitators to primary and secondary melanoma prevention, discussions were often more generally focused on skin cancer given low baseline knowledge about melanoma in particular.

Nonetheless, the use of focus group format for this exploratory study provided rich data that may have not been well obtained via other methods.

## 5 CONCLUSION

Our large, qualitative analysis of stratified groups provides increased understanding of unique barriers and facilitators of melanoma awareness, prevention, and early detection, health care, and cultural and health literacy needs between low‐income NLW and Latinx participants in distinct rural geographies across California. Our findings enrich existing data regarding inequities in later‐stage presentation among communities of color and lower SES groups. Engaging trusted community members and health workers in raising melanoma awareness may be conducive to increasing skin examination practices and improving treatment navigation within health systems. These elements are critical to improve melanoma early diagnosis in communities at highest risk of fatal melanoma in California and other states with similar socio‐demographics. Public health efforts can be designed to incorporate appropriate educational resources that compliment community health navigation in rural and lower SES areas and utilize teledermatology to increase specialist access. Our findings can support the delivery of more effective primary and secondary melanoma prevention for underserved populations across geographic regions.

## AUTHOR CONTRIBUTIONS

**Rachel J. Mesia:** Conceptualization (supporting); data curation (equal); formal analysis (equal); investigation (equal); methodology (supporting); project administration (equal); visualization (equal); writing – original draft (equal); writing – review and editing (equal). **Patricia Rodriguez Espinosa:** Formal analysis (supporting); visualization (equal); writing – original draft (equal); writing – review and editing (equal). **Hayden Hutchison:** Formal analysis (supporting); investigation (equal); project administration (equal); writing – original draft (supporting); writing – review and editing (equal). **Nadia Safaeinili:** Conceptualization (supporting); data curation (equal); formal analysis (equal); investigation (equal); methodology (supporting); visualization (supporting); writing – original draft (equal). **Laurel J. Finster:** Formal analysis (supporting); investigation (supporting); writing – review and editing (supporting). **Vijaytha Muralidharan:** Writing – review and editing (supporting). **Beth A. Glenn:** Formal analysis (supporting); writing – review and editing (supporting). **Robert W. Haile:** Conceptualization (equal); formal analysis (supporting); investigation (supporting); methodology (equal); writing – review and editing (supporting). **Lisa Goldman Rosas:** Conceptualization (equal); investigation (supporting); methodology (equal); writing – review and editing (supporting). **Susan M. Swetter:** Conceptualization (equal); funding acquisition (lead); investigation (supporting); methodology (equal); writing – original draft (equal); writing – review and editing (equal).

## FUNDING INFORMATION

Mary E. Brenneisen Fund at Stanford Medicine and in part, National Center for Advancing Translational Sciences of the National Institutes of Health, Award Number UL1TR003142. This material is the result of work supported with resources and the use of facilities at the VA Palo Alto Health Care System, Palo Alto, California.

## CONFLICT OF INTEREST

There authors declare no conflicts of interest.

## Data Availability

The data that supports the findings of this study are available in the supplementary material of this article.
